# Tipping the Balance of Autism Risk: Potential Mechanisms Linking Pesticides and Autism

**DOI:** 10.1289/ehp.1104553

**Published:** 2012-04-25

**Authors:** Janie F. Shelton, Irva Hertz-Picciotto, Isaac N. Pessah

**Affiliations:** 1 Graduate Group in Epidemiology, Department of Public Health Science, University of California, Davis, Davis, California, USA; 2 UC Davis Medical Investigation of Neurodevelopmental Disorders (MIND) Institute, Sacramento, California, USA; 3 Department of Molecular Biosciences, University of California, Davis, Davis, California, USA

**Keywords:** autism spectrum disorders, carbamate, gene–environment interaction, immune, mitochondria, neuroexcitation, organochlorine, organophosphate, oxidative stress, pesticide, pyrethroid

## Abstract

Background: Autism spectrum disorders (ASDs) have been increasing in many parts of the world and a portion of cases are attributable to environmental exposures. Conclusive replicated findings have yet to appear on any specific exposure; however, mounting evidence suggests gestational pesticides exposures are strong candidates. Because multiple developmental processes are implicated in ASDs during gestation and early life, biological plausibility is more likely if these agents can be shown to affect core pathophysiological features.

Objectives: Our objectives were to examine shared mechanisms between autism pathophysiology and the effects of pesticide exposures, focusing on neuroexcitability, oxidative stress, and immune functions and to outline the biological correlates between pesticide exposure and autism risk.

Methods: We review and discuss previous research related to autism risk, developmental effects of early pesticide exposure, and basic biological mechanisms by which pesticides may induce or exacerbate pathophysiological features of autism.

Discussion: On the basis of experimental and observational research, certain pesticides may be capable of inducing core features of autism, but little is known about the timing or dose, or which of various mechanisms is sufficient to induce this condition.

Conclusions: In animal studies, we encourage more research on gene × environment interactions, as well as experimental exposure to mixtures of compounds. Similarly, epidemiologic studies in humans with exceptionally high exposures can identify which pesticide classes are of greatest concern, and studies focused on gene × environment are needed to determine if there are susceptible subpopulations at greater risk from pesticide exposures.

Causes for the recent rise in autism diagnoses throughout the United States remain largely unknown. In California, a 600% increased incidence in autism was observed among children up to 5 years of age for births from 1990 to 2001, yet only one-third of the rise could be explained by identified factors such as changing diagnostic criteria and a younger age at diagnosis (Hertz-Picciotto and Delwiche 2009). Across the United States, autism spectrum disorders (ASD) are now estimated to affect 1 in 88 eight-year-olds, with much higher prevalence in boys (1 in 54) than girls (1 in 252) (Centers for Disease Control and Prevention 2012). Autism is a heterogeneous, behaviorally defined condition often diagnosed in children prior to age 3 years. Although each individual diagnosis must meet specific criteria related to deficits in social interaction and language and to the presence of repetitive behaviors or restricted interests, autism phenotypes vary widely, even among concordant twins (Le Couteur et al. 1996).

Idiopathic autisms are diagnosed 4–5 times more often in boys than girls and frequently involve a wide range of genes that confer susceptibility as opposed to a singular heritable factor (Geschwind 2011). Genetic contributions to autism risks involve rare mutations, complex gene × gene interactions, and copy number variants (CNVs) including deletions and duplications (Stankiewicz and Lupski 2010). In a recent series of papers, rare *de novo* point mutations were associated with autism in parent–child trios with sporadic ASD (Neale et al. 2012; O’Roak et al. 2012; Sanders et al. 2012), and those mutations were more frequently derived from fathers, increasing with paternal age (O’Roak et al. 2012). Although twin studies have demonstrated evidence of heritability—a stronger concordance among monozygotic than dizygotic twins (Bailey et al. 1995; Rosenberg et al. 2009; Steffenburg et al. 1989)—in a recent twin study that parsed the contribution from genetics versus the environment, a larger component of the risk of autism was attributable to environmental factors than genetics alone (e.g., Hallmayer et al. 2011). The genetic and twin studies of autism point to variability unexplained by heritable factors and, in recent years, associations between gestational pesticide exposures and ASD or behaviors that are characteristic of pervasive developmental disorders have been reported.

Using exposure estimates from a historical pesticide use database, a study of mothers living in the California Central Valley showed that children born to mothers who had been exposed to organochlorine (OC) insecticides that were agriculturally applied within 500 m of the home between gestational days (GD) 26 and 81 (during neural tube closure) were 7.6 times more likely to be diagnosed with ASD than the children of mothers who lived in the lowest exposure quartile. Associations were also observed for the pyrethroid insecticide bifenthrin and for the organophosphate (OP) chemical class when comparing the cumulative exposure over the course of gestation among the highest versus lowest quartile (Roberts et al. 2007). Although Roberts et al. (2007) present provocative preliminary data and higher odds at closer proximity (a dose–response relationship), unmeasured confounding could have occurred for other exposures such as prenatal vitamin intake or occupational exposures. Additionally, because cases were obtained from the Department of Developmental Services (DDS) and controls from the birth certificate registry, misclassification of cases and controls may have occurred as children who receive an early diagnosis of autism are sometimes reclassified at a later date, and controls may include children who are on the autism spectrum but have not received a DDS diagnosis.

In a prospective cohort study also from the California Central Valley, a 230% increase in maternally reported symptoms of pervasive developmental disorders (PDD) was observed per 10-nM/L increase in prenatal maternal urinary levels of OP metabolites (Eskenazi et al. 2007). PDD is the greater diagnostic umbrella under which ASD falls, also encompassing Rett Syndrome, childhood disintegrative disorder (CDD), and pervasive developmental disorder–not otherwise specified (PDD-NOS). Although the prospective study design has the benefit of accuracy in exposure ascertainment from biospecimens collected during pregnancy, it is generally not feasible to obtain a cohort large enough to observe enough cases of full syndrome autism. Consequently the broader definition of borderline PDD increases the numbers but lacks specificity. Although these studies are by no means conclusive in establishing an autism–pesticide association, they do raise important questions regarding the health effects of these compounds on the developing fetus. In light of these findings and the current theories of autism pathophysiology, we review here potential pathways by which gestational pesticide exposure might contribute to autism, linking what is known about the origins of autism with information on biological effects of pesticides to generate clearer hypotheses that can help guide future research in this area.

## Pesticide Exposure in the General Population

**Figure 1:**
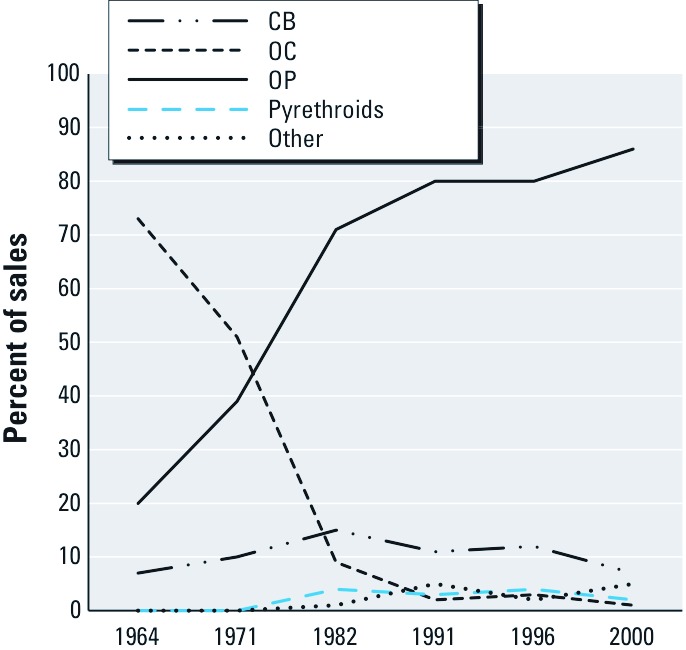
Agricultural pesticide trends in the United States by percent of sales, 1964–2000 (U.S. Department of Agriculture 2006).

Pregnant women are exposed to pesticides through a wide variety of sources. Although many of the mechanisms of action outlined here have been observed in association with higher exposures than are likely to occur in the general population, it is difficult to estimate the direct dosage to a pregnant woman who may be applying pesticides in or around her home or to her pets, consuming food with residues of pesticides and pesticide metabolites, and inhaling air from agricultural or urban spraying near her home and workplace. Moreover, urine and blood levels indicate exposure to pregnant women is widespread. In the 2003–2004 National Health and Nutrition Examination Survey (NHANES), which recruits a representative sample of the U.S. adult population, 83% of pregnant women had detectable levels of urinary dimethylthiophosphate, an OP metabolite [geometric mean (GM), 2.43 µg/L urine]. DDE, the breakdown product of the persistent OC pesticide DDT, was detected in 100% of pregnant women with a GM of 140.4 ng/g lipid (Woodruff et al. 2011). Trends in pesticide use in the United States since 1964 have shown steep increases in the use of OPs, which make up the vast majority of pesticide sales, and rapid decreases in OC use following the 1972 ban on DDT (Figure 1). More recently, as OPs have been banned for residential uses, pyrethroid sales have increased rapidly (Williams et al. 2008).

## The Quest for Animal Models of Autism and Environment

A variety of animal models have been developed that aid in understanding the mechanisms that may induce one or several of the core features of autism (Ey et al. 2011; Hamilton et al. 2011; Tabuchi et al. 2007). In particular, transgenic and knock-in mouse lines with targeted anomalies in genes associated with autism and the development of a comprehensive set of rodent assays to assess social interaction, communication, and repetitive behaviors, have greatly enhanced our ability to test hypotheses about the causes of autism (Silverman et al. 2010). However, implementations of these tools toward understanding gene × environment interactions that promote impairments in the three key behavioral domains have lagged. The *Shank3* (SH3/ankyrin domain gene 3) (Peca et al. 2011) and oxytocin knockout mice (Crawley et al. 2007) are examples of monogenetic insults that disrupt all three domains. However, because only a small proportion of autism cases result from complete loss of a single gene, knockout animal models may not be as useful as models that carry mutations that impart partial gain or loss of gene function.

Functional impairments as seen in the reeler mouse (Laviola et al. 2009) and Timothy syndrome mouse models (Bader et al. 2011) are more relevant to the multi-gene and environment model of autism risk. In a subsection of a paper describing the paradoxical effects of acetylcholinesterase [AChE; the enzyme responsible for hydrolyzing the neurotransmitter acetylcholine (ACh)] in the reeler mouse, Laviola et al. (2006) describe the complexity of a gene × environment model whereupon exposure to chlorpyrifos restored behaviors to near normal that were initially impaired in the homozygous reeler mouse, and partially impaired in the heterozygous reeler mouse. It was shown that deficient cholinergic transmission in reeler mice could be restored by chlorpyrifos-mediated AChE inhibition. Subsequent studies found that perinatal estradiol levels influence the number of Purkinje cells and were regulated by reelin levels (Biamonte et al. 2009; Sigala et al. 2007). This sex × gene × environment interaction model serves more readily as a clue for further epidemiologic follow-up to understand autism etiology in humans (Halladay et al. 2009).

Several autism-associated genes are involved in Ca^2+^ signaling and regulation (Halladay et al. 2009; Pessah and Lein 2008). The Timothy syndrome mouse model of autism involves a single nucleotide mutation essential for proper voltage-dependent inactivation of the pore-forming subunit of the L-type calcium channel Ca_v_1.2 (Splawski et al. 2004). Ca_v_1.2 has been proposed to play direct roles in the development of synaptic plasticity (Morgan and Teyler 1999) and in gene translation and transcription (Dolmetsch 2003; Lenz and Avruch 2005; West et al. 2002).

Ca^2+^ signaling can be disrupted by polychlorinated biphenyls (PCBs, which are employed in a wide variety of industrial uses) (Pessah et al. 2010), the OC pesticides lindane and dieldrin (Heusinkveld and Westerink 2012), and several types of pyrethroid pesticides (Soderlund 2012). In a study comparing physiological effects of 11 pyrethroid compounds in rats, the type 2 pyrethroids strongly induced increased Ca^2+^ channel influx into the cell, whereas the type 1 pyrethroids did not (Breckenridge et al. 2009). It should be noted that these three exposure types induced calcium perturbations at levels below those described as having a toxic effect on the basis of primary mechanisms of action.

One could argue that mouse, rat, or zebrafish models may not demonstrate the core deficit that sets autism apart from other developmental disorders: a lack of social reciprocity. Recently, the prairie vole has been cited as a better model of autism due to its high degree of socialized behavior. For example, male prairie voles demonstrated social withdrawal after 10 days of dietary exposure to mercury, indicating a sex-specific effect of the exposure which induced a unique attribute of autism, social avoidance (Curtis et al. 2010).

## Excitation/Inhibition Dysregulation of Neuronal Development

Rubenstein and Merzenich (2003) elegantly described a model of autism whereby the cortical networks that govern language and social behavior are skewed toward increased excitation (i.e., away from inhibition), resulting in an overall hyperexcitable state. Their hypothesis addresses both genomic and environmental factors influencing glutamate and GABA (γ-aminobutyric acid)–mediated neurotransmission, resulting in more noise in neural networks.

By poundage applied, the majority of pesticides inhibit AChE. Examples include two of the most frequently used pesticides worldwide, the OPs chlorpyrifos and diazinon, as well as the monomethyl carbamates (CBs), including propoxur and methomyl. Insecticides that target voltage-gated sodium channels (e.g., pyrethroids and DDT), the nicotinic ACh receptors (nAChR) themselves (e.g., imidacloprid), and GABA_A_ receptors (e.g., OC and fipronil) are ranked next highest in use in overall poundage applied (Casida 2009). The levels of ACh- and GABA-mediated neurotransmission and the activity of voltage-dependent sodium channels are critical throughout prenatal and postnatal development, defining the ratio of excitatory and inhibitory neurotransmission in the brain, but also promoting and refining neural networks in the developing and adult brain (Belmonte and Bourgeron 2006).

*GABA signaling pathways.* GABA is critical for normal development and regulation of neurotransmission (Campbell 1996). GABA activates two major families of receptors expressed in the mammalian brain: *a*) GABA_A_ and GABA_C_ receptors that promote chloride fluxes, and *b*) GABA_B_ receptors that are coupled to G-protein signaling. In adults, GABA_A_ receptor activation promotes chloride influx and hyperpolarization of the membrane, decreasing neuronal excitability. However, during fetal development, the chloride gradients across the membrane are reversed, and therefore activation of GABA_A_ receptors in the hippocampus and neocortex causes net chloride efflux and enhanced excitation (Watanabe et al. 2002). Thus, the temporal expression and spatial localization of GABA receptors within the brain can determine the patterns and activity of neural circuits. Numerous subunit isoforms for the GABA_A_ receptor are developmentally regulated during the perinatal period and have distinct biophysical and pharmacological properties that contribute to their physiological (Cossart et al. 2005) and pathophysiological (Stafstrom et al. 2010) functions. GABA is known to regulate many aspects of neural stem cell proliferation, differentiation, migration, and elongation (Varju et al. 2001). Because of observed deficits in social and exploratory behavior, the GABA_A_ receptor β3-gene–deficient mouse has been suggested as an animal model of autism spectrum disorder (DeLorey et al. 2008).

Disruptions in the GABA system have been reported to be associated with autism in studies of receptor density from brain tissue (Blatt et al. 2001) as well as in studies of genetic association (Buxbaum et al. 2002; Cook et al. 1998; McCauley et al. 2004). In postmortem cerebellar tissue samples from the brains of adults with autism, relative numbers of GABA_A_ receptors were reduced in 4 cases as compared with 8 controls, and GABA_B_ expression was altered in 5 cases as compared with 7 controls (Fatemi et al. 2009a, 2009b). Decreased expression of GABA_A_ receptor β3 was shown to be associated with *MECP2* (the gene for methyl CpG binding protein 2) impairment in brain tissue samples from cases of autism, Angelman syndrome, and Rett syndrome (Samaco et al. 2005). In a family-based study, single nucleotide polymorphisms were examined in 470 families with at least one case of autism (266 multiplex, 204 triads) for GABA subunits on 14 alleles. Findings showed significant associations for GABA_A_ receptor polymorphisms, in particular the A4 subunit and gene × gene interaction between receptor subunits (Ma et al. 2005).

In rats, prenatal exposure to the OC pesticides dieldrin and lindane reduces GABA_A_ receptor binding capabilities in the brainstem (Brannen et al. 1998). In another rat study, prenatal dieldrin exposure was found to alter mRNA expression and subunit composition of GABA_A_ receptors (Liu et al. 1998). Results from *in vitro* cortical neuronal cultures have shown endosulfan and related OC pesticides to increase Akt phosphorylation, an effect mediated by the activation of ERβ, and to activate ERK1/2 through a mechanism involving GABA_A_ and glutamate receptors (Briz et al. 2011). In humans, a diminished ability to bind GABA contributes to poor muscle tone, which is observed in over half of persons with autism (Ming et al. 2007), and induces hyperexcitable states as seen in epilepsy, a comorbidity in approximately 20% of autistic cases (Bolton et al. 2011; Tuchman and Cuccaro 2011).

PCBs are OCs that had broad industrial uses, including use as adjuvants in paints and pesticide formulations (U.S. Environmental Protection Agency 2011). Although banned approximately 40 years ago, PCB exposures remain a concern to human health because of their persistence in the environment. Developmental and *in vitro* studies in rodents and nonhuman primates have demonstrated the ability of non-coplanar PCBs to cause imbalances in excitatory and inhibitory neurotransmission within critical regions for language development (Kenet et al. 2007), social cognition (Nakagami et al. 2011), and seizures (Kim and Pessah 2011; Kim et al. 2009). A substantial body of epidemiologic literature has provided evidence that cognitive deficits are associated with elevated PCB exposures, and more recently, elevated prenatal exposures to mono-*ortho* PCBs were found to be predictive of lower scores on both the Mental Development Index (MDI) and the Psychomotor Development Index (PDI) of the Bayley Scales of Infant Development (Park et al. 2010). Furthermore, an analysis of seven hydroxylated metabolites of PCBs in cord blood revealed that the metabolite from mono-*ortho* substituted PCBs were the only ones associated with reduced MDI and PDI scores (Park et al. 2009). These findings underscore the complexity of toxicities within a compound class and, by the same principle, the critical need to characterize differences among, for example, OPs or pyrethroids.

*ACh-signaling pathways.* ACh-mediated neurotransmission is widely involved in the development of both the peripheral and the central nervous systems, and continues to play a critical role in regulating muscle movement, learning, attention, cognition, and memory throughout adulthood. ACh regulates aspects of nerve excitation and inhibition that influence brain plasticity, arousal, and reward. ACh increases excitation both directly and indirectly, and works through both nicotinic and muscarinic receptors to stimulate inhibitory interneurons, thereby modulating the activity of downstream effectors in a complex manner (Brown 2010; Scharf 2003).

Several cholinergic abnormalities have been reported in autism [Bauman and Kemper 2005; Perry et al. 2001; reviewed by Deutsch et al. (2010)]. In brief, studies of postmortem brain tissue have reported reduced nAChR binding in the frontal and parietal cortices (comparing 7 cases with 10 controls), reduced M1-muscarinic receptor binding in the parietal cortex (comparing 5 cases with 5 controls), and increased concentration of brain-derived neurotrophic factor (BDNF) (comparing 5 cases with 5 controls) (Deutsch et al. 2010). (BDNF is involved in the development and function of cholinergic neurons.) Although these studies involved small sample sizes, they suggest cholinergic abnormalities may be present in persons with autism.

OP insecticides irreversibly inhibit the active site of AChE, and while the severity of neurodevelopmental effects in animal studies correlate with AChE inhibition, additional neurotoxic effects have been observed at concentrations below the level sufficient to induce enzyme inhibition (Eddins et al. 2010; Levin et al. 2003; Slotkin et al. 2008). These effects include altered cell packing density, decreases in serotonin receptor and nAChR levels (Levin et al. 2010), altered Ca^2+^ and K^+^ ion concentrations (Harrison et al. 2002; Murgia 2004), and oxidative stress (Aluigi et al. 2005). Metabolism of OPs is mediated by the paraoxonase1 enzyme (PON1), whereby fast metabolizers suffer less AChE inhibition than slow metabolizers in response to the same level of exposure (Costa et al. 2005).

Pertinent to the male predominance observed in autism, sex selective developmental effects have been seen in animal models exposed to OPs. Chlorpyrifos exposure (1 mg/kg/day) in rats during postnatal days (PND) 1–4 decreased the number of errors in working and reference memory made by females, but increased the number of such errors made by males. These effects persisted into adolescence and adulthood, indicating a long-term consequence of exposure (Levin et al. 2001). Another study in rats showed that developmental exposures to low doses of the OP parathion induced greater developmental deficits in spatial navigation and working memory among males than females (Levin et al. 2009). Although these behaviors are not core features of autism, these findings provide evidence of different effects of early exposures between the sexes. In addition, parathion administration on PND1–4 at levels that barely inhibited cholinesterase was associated with deficits at 14–19 months of age, showing these deficits worsen with age (Levin et al. 2009).

The ability of OPs to inhibit AChE varies dramatically by chemical structure, which also determines reversibility. Aluigi et al. (2005) conducted a study examining the AChE-mediated developmental effects of OP exposure on chick embryos and discovered that 10^–6^ M chlorpyrifos was sufficient to inhibit head development. Even lower concentrations of chlorpyrifos-oxon disrupt axonal growth of rat dorsal root ganglia neurons (Yang et al. 2008), and sensory neuron development in zebrafish (Yang et al. 2011), indicating that exposure to very low levels of this OP has the potential to adversely influence development of neural networks (Yang et al. 2011). Persistent neurobehavioral consequences of chlorpyrifos exposure in zebrafish have also been demonstrated (Eddins et al. 2010; Levin et al. 2003). Although chlorpyrifos is still used worldwide in residential settings, residential use has been banned in the United States because of its neurotoxicity. However, no restrictions have been placed on its agricultural use.

## Oxidative Stress and Mitochondrial Dysfunction

Cellular energy production through the degradation of ATP by mitochondria is necessary for muscle development and brain function. Mitochondrial dysfunction has three major consequences: *a*) decreased ATP production, *b*) increased production of reactive oxygen species (ROS) and oxidative damage, and *c*) induction of apoptosis (Rossignol and Frye 2012). These biochemical changes have been implicated in autism and can also be induced by exposure to OP, OC, and CB pesticides (Franco et al. 2009; Karami-Mohajeri and Abdollahi 2011; Rohlman et al. 2010). Although multiple modes of action have been described for specific organohalogens and halogenated insecticides, many induce dysregulation of Ca^2+^-mediated signaling and production of mitochondrial ROS (Mariussen and Fonnum 2006). A thorough mechanistic hypothesis of autism via genetic risk and oxidative stress has been described by Deth et al. (2008).

Nearly all insecticides discussed in this review induce oxidative stress. Permethrin, a pyrethroid used in agriculture and in topical creams for lice and scabies induces oxidative stress and apoptosis in the nervous system of zebrafish (Shi et al. 2011). Malathion, an OP commonly used in aerial spraying throughout the 1980s for the Mediterranean fruit fly and more recently to control mosquito vectors of West Nile Virus, induces mitochondrial dysfunction in liver cells at low concentrations and cytotoxicity at higher concentrations (Moore et al. 2010). The OC insecticide methoxychlor has been shown in mice to inhibit brain mitochondrial respiration (Schuh et al. 2005) and to cause mitochondrial dysfunction and oxidative damage in the mouse ovary (Gupta et al. 2006). More recently, methoxychlor-mediated mitochondrial dysfunction was found to cause oxidative damage and dysfunction of the dopamine system in brains of mice (Schuh et al. 2009). Another study examining the effect of the OP dichlorvos on rat brain mitochondria found that chronic, low-level exposure can cause mitochondrial disruption and apoptosis of neuronal cells via the release of cytochrome c and activation of caspase 3 after *in vitro* exposure (Kaur et al. 2007). Developmental exposure to the OP chlorpyrifos can permanently decrease dopamine levels in zebrafish into adulthood (Eddins et al. 2010), which is important to note in the context of an already disrupted dopamine system in autism (Muhle et al. 2004).

Several studies have shown that the toxicity of pyrethroid insecticides, many of which are organohalogen derivatives, is mediated by both the dysregulation of cytoplasmic Ca^2+^ signaling and the induction of oxidative stress (Cao et al. 2010; Kale et al. 1999; Soderlund 2012; Yan et al. 2011; Zhang et al. 2010). After the ban on residential uses of chlorpyrifos, household OP insecticides have been replaced with other insecticides, namely pyrethroids and fipronil, a phenylpyrazole insecticide. A comparative toxicity study was conducted on rat PC12 cells to evaluate the hypothesis that fipronil is less toxic than chlorpyrifos, but fipronil was found to induce higher oxidative stress than chlopyrifos, an effect that was not mediated by the GABA_A_ pathway (Lassiter et al. 2009).

Although the role of mitochondrial function in the autistic phenotype is not fully understood, approximately 8% of ASD cases experience mitochondrial dysfunction, compared with 0.05% of the general population [reviewed by Haas (2010)]. Mitochondrial dysfunction and increased mtDNA over-replication and mtDNA deletions were reported more frequently in lymphocytes from 10 children with autism as compared with lymphocytes from 10 typically developing controls (Giulivi et al. 2010).

## Immune Toxicity

Prenatal disruption of immune development can result in atopy, allergy, deficits in immune competence, and autoimmunity in early childhood (Hertz-Picciotto et al. 2008). Recent studies on intestinal flora have shown the immune system is highly involved and inextricably linked with neurodevelopment and subsequent behavior (Diamond et al. 2011; Heijtz et al. 2011). In turn, the immune response can also be strongly influenced by neurochemistry (Diamond et al. 2011). Children with autism experience a wide array of immune abnormalities. Recent reviews on this topic report altered cytokine profiles, altered cellular immunity, low levels of lymphocytes and T-cell mitogen responses, neuroinflammation, and autoantibodies directed at nuclear proteins (Ashwood et al. 2006; Goines and Van de Water 2010). Reduced levels of IgG and IgM have also been reported, which were correlated with a higher prevalence of aberrant behavioral symptoms in a study of 271 children with autism or developmental delay or who were typically functioning (Heuer et al. 2008). In a comparison of plasma cytokine levels from children with autism (*n* = 97) and typically developed controls (*n* = 87), cases had higher levels of proinflammatory cytokines compared with neurotypical children, and the concentrations of cytokines corresponded with impaired behavioral outcomes in a dose–response fashion (Ashwood et al. 2011).

Exposure to several types of pesticides may result in decreased immune competence, immune enhancement, and/or autoimmunity (Corsini et al. 2008). OPs are particularly immunotoxic (Galloway and Handy 2003) and have been shown to suppress natural killer cells, lymphokine-activated killer cells, and cytotoxic T lymphocytes by inhibiting granzymes, impairing the FasL/Fas pathways, and inducing apoptosis of immune cells (Li 2007). Pyrethroids have also been shown to be immunotoxic in animal models. Rats treated subchronically with permethrin showed large increases of superoxide anion production and hydrogen peroxide–myeloperoxidase activity in polymorphonuclear neutrophils (Gabbianelli et al. 2009). These effects were demonstrated not only for permethrin, but also for its major metabolites.

Insecticide exposures can induce inflammatory or suppressive immunological effects depending on the compound and the immunological outcome in question. Gestational exposure of rats to atrazine, an endocrine-disrupting triazine herbicide, demonstrated immunosuppressive effects [specifically, decreased delayed-type hypersensitivity (DTH) and antibody production] in male offspring only (Rooney et al. 2003). In a study of both male and female mice, gestational exposure to atrazine at nontoxic, environmentally relevant doses administered from GD14 to PND21, was associated with decreased socialization behaviors and changes in exploratory behavior, with males displaying feminized behavioral profiles (Belloni et al. 2011).

Neuroinflammation has been observed in the postmortem brain tissue of persons with autism across several age ranges (Li et al. 2009; Morgan et al. 2010; Vargas et al. 2005). Chlorpyrifos, an OP banned for residential use in 2002, and cyfluthrin, a type 2 pyrethroid used to replace chlorpyrifos, were compared for toxicological and toxicogenomic effects to primary human fetal astrocytes. Cyfluthrin had equivalent or more toxic effects in most assays, and up-regulated several insulin related genes and proinflammatory genes on the IFN-γ (interferon-γ) pathway, including *IL6R* (the gene for the interleukin 6 receptor) and *GFAP* (the gene for glial fibrillary acidic protein). Additionally, both compounds were found to promote inflammatory activation of astrocytes. The authors suggested that the combination of increased insulin production and inflammation could lead to a state of chronic brain inflammation that might significantly alter brain development (Mense et al. 2006).

Taken together, these studies indicate that gestational exposure to pesticides can induce immunological abnormalities as well as behavioral abnormalities. It is possible that the neurodevelopmental and the immune abnormalities observed in autism are downstream manifestations of the same underlying process given the tightly regulated interconnection between the developing systems in utero. The role of the immune phenomena as a cause, effect, or side effect of autism was recently reviewed and was postulated to be in part causal (Onore et al. 2012). In addition to autism, schizophrenia and major depressive disorders have also been noted to be accompanied by perturbations of the immune system, recently reviewed in an extensive monograph (Patterson 2011).

## Parental Thyroid Hormone Levels and Brain Development

Adequate levels of *in utero* thyroid hormones are critical for brain development. Maternal thyroid impairment has been suggested as an underlying mechanism for developmental impairments resulting from exposures to environmental chemicals such as PCBs and polybrominated diphenyl ethers (PBDEs) used as flame retardants (Winneke 2011). Pesticides have been found to interfere with thyroid function by preventing iodine uptake [e.g., mancozeb, thiocyanates, 2,4-D (2,4-dichlorophenoxyacetic acid)] and peroxidation (e.g., aminotriazole, endosulfan, malathion), and by preventing the conversion of thyroxine (T_4_) to triiodothyronine (T_3_) (e.g., aminotriazole, dimethoate, fenvalerate) (Colborn 2004). In a review of the effects of mild-to-moderate iodine deficiency in humans, diminished maternal T_4_ was associated with disorders of mental and/or psychomotor development (Zimmermann 2007).

Roman (2007) hypothesized that even transient intrauterine deficits in thyroid hormones (as little as 3 days) at critical points in gestation could alter the cortical architecture, interfering with neuronal migration and Purkinje cell growth, indications of both of which have been observed in autopsy studies of autism (Fatemi et al. 2002; Wegiel et al. 2010). Because the human fetus does not start producing sufficient thyroid hormones until gestational week 18 (Burrow et al. 1994), adequate maternal thyroid hormones are critical to neurodevelopment in early fetal life, particularly for reelin-regulated neuronal migration (Pathak et al. 2011). Additionally, sex-mediated effects have been observed after exposure to chlorpyrifos on GD17–20, with the induction of increased levels of free T_4_ in female but not male mice (Haviland et al. 2009).

## Vulnerable Genetic Subpopulations

The primary neurological targets of commonly used insecticides (Scharf 2003) can be paired with vulnerable genetic subpopulations that may be at increased risk for autism (Table 1). Because of both the large number of genetic alterations and gene × gene interactions that have been implicated in autism, and the phenotypic heterogeneity in cases, the notion that a single environmental exposure can be to blame for the majority of cases is unrealistic. Also, because the dosage of pesticides to nonoccupationally exposed women is likely to be lower than that required to induce the mechanisms of injury observed in many animal models, genetic susceptibility becomes a critical factor in this discussion.

**Table 1:** Insecticide compounds with a generalized excitatory neurological effect.

Primary neurological target	Insecticide class	Mode of action	Vulnerable genetic subpopulations
AChE		OP		Inhibition		PON1 polymorphisms
		CB		Inhibition		
Voltage-gated sodium channel		OC		Modified gating kinetics		SCN1A, SCN1B
		Pyrethrin/pyrethroid		Modified gating kinetics		HCE1 (CES1)
						HCE2 (CES2)
GABA-gated chloride channel		Cyclodienes (a form of OC)		Antagonism		GABA receptor polymorphisms
		Phenylpyrazole		Antagonism		
nAChRa		Neonicotinoid		Agonism		Haploinsufficiency of α7 nAChR
Adapted from Scharf (2003). Abbreviations: HCE1 (CES1)/HCE2 (CES2), human carboxylesterase 1/2 (human cholesterase 1/2); SCN1A/SCN1B, sodium channel, voltage-gated channel protein, type 1 alpha/beta. aNeonicotinoids first induce excitation, which is followed by inhibition.						

In 2001, the reelin gene was implicated in autism risk when repeats (11+) in the 5´ untranslated region were associated with 72% transmission to affected siblings and only 32% transmission to unaffected siblings (Persico et al. 2001). The proteolytic activity of reelin on extracellular matrix proteins that control neuronal migration is significantly inhibited by OP pesticides (Sinagra et al. 2008), and OP metabolism efficiency is regulated by the gene for paraoxonase 1 (*PON1*) (Mackness et al. 1997). Interestingly, an association between less active forms of the *PON1* gene and autism was observed in Caucasian families in North America, but not in Italian families, leading authors to hypothesize that the slow metabolizing polymorphism confers risk in areas with high levels of OPs but may not affect autism risk otherwise (D’Amelio et al. 2005).

## Conclusions

We have reviewed several mechanisms by which pesticides may increase the risk of autism, summarized in Table 2. Pesticides may or may not, however, play a role in the trend of increasing autism prevalence, which itself is likely due to a confluence of multiple phenomena, including changes in diagnostic practices, physician and lay awareness, the availability of treatments, and the prevalence of a variety of environmental chemical, medical, and food-related exposures. While pesticide use patterns have changed, home and ambient environments also include other exposures that have changed over time as a result of regulatory and economic factors (e.g., flame retardants, plasticizers, solvents, stabilizers, antimicrobials).

**Table 2:** Mechanisms by which gestational exposure to certain classes of pesticides may induce observed pathophysiologic symptoms of autism.

Mechanism of action/ Route to autism pathophysiology	Observed effects	Specific pesticides	Class of pesticide	Reference
Developmental neurotoxicity								
Alteration of excitation/inhibition mechanisms		Decrease in GABA receptors		Dieldrin (prenatal exposure in rats)		OCs		Brannen et al. 1998; Liu et al. 1998
		Inhibition of GABA		General function of OC, pyrethroid pesticides		OC, pyrethroid		
		Inhibition of AChE		General function of OP, CB pesticides		OPs, CBs		
Mitochondrial dysfunction								
Oxidative stress		Apoptosis of neuronal cells		Dichlorvos (rat brain)		OPs		
		Inhibition of mitochondrial respiration		Methoxychlor (mice brain)		OCs		Kaur et al. 2007; Schuh et al. 2005
Immune toxicity								
Immunosuppression		Decreased DTH and antibody production		Atrazine (gestational exposure to rats)		Triazine		Rooney et al. 2003
Neuroinflammation		Activation of human fetal astrocytes, increased expression of proinflammatory cytokines		Cyfluthrin, chlorpyrifos (primary human fetal astrocytes)		Pyrethroid, OPs		Mense et al. 2006
Maternal hypothyroxinemia								
Insufficient gestational thyroid hormones		Decreased T4, inhibition of T4 deiodination to T3, prevention of iodine uptake		Acetochlor, alachlor, mancozeb, thiocyanates, 2,4-D, aminotriazole, endosulfan, malathion (multiple animal studies)		OCs, thiocyanates, OPs		Cheek et al. 1999; Colborn 2004; Goldner et al. 2010; Rathore et al. 2002


Pesticides are composed of a parent product, inert ingredients, and in some cases agonists that enhance the functionality of the parent compound, and all of these ingredients may be degraded to metabolites that also distribute throughout the body. Consequently, pesticide formulations represent a mixture of compounds that might contribute to observed effects. Difficulties in distinguishing the effects of metabolites versus parent compounds may have confounded associations observed in many studies of urinary metabolites and neurodevelopment, and very few studies have examined the main effects or effect modification of exposure to piperonyl butoxide, which slows the metabolism of several types of pesticides by inhibiting cytochrome P450 enzymes.

Although pesticides are a biologically plausible contributor to autism, research in several critical areas is needed to understand cognitive and behavioral consequences of gestational exposure in humans. First, animal studies suggest critical windows of exposure, yet in humans the window or windows of biologic susceptibility remain unknown, and would be expected to vary by mechanism. Second, studies of nontoxic, environmentally relevant doses are needed to understand the effects of developmental neurotoxicity in the context of a background of genetic susceptibilities. Third, the vast majority of exposures occur in combination with exposures to other ubiquitous and/or persistent compounds such as flame retardants, plasticizers, and other pesticides. More research on combinations of exposures may reveal interactions between environmental exposures, such as effect modification by chemical additives to pesticide compounds. In light of the recently revised prevalence estimates of autism (1 in 88), large birth cohorts, such as the National Children’s Study (NCS), which aim to enroll women at pregnancy and follow the children over time, are well positioned to obtain enough cases and to examine prenatal exposures prospectively. Pending accurate and reliable exposure estimates in critical time windows, and enrollment of approximately 100,000 children resulting in 1,000 or more cases of autism, NCS can contribute greatly to our understanding of these associations. Finally, more case–control studies with large populations of participants with confirmed diagnoses of autism that examine environmental exposures in relation to severity of the core domains of language impairment, social avoidance, and repetitive behaviors or insistence on sameness may shed light on possible exposure-related endophenotypes.

Although we have described several possible avenues by which pesticide exposure may influence autism, the dearth of studies on large occupational and pregnancy cohorts with adequate exposure assessment impedes our understanding of *a*) whether pesticides are consistently associated with autism risk, and *b*) if so, which pesticide compounds and which components of those compounds might actually contribute to autism risk. Grandjean and Landrigan (2006) hypothesized that our exposure to chemicals that have not been adequately tested for developmental neurotoxicity has led to a silent pandemic. Further research is warranted to provide the evidence base that can ultimately lead to reducing or eliminating these potentially damaging exposures through changes to regulatory policy, consumer behavior, or dietary choices.

## References

[r1] Aluigi MG, Angelini C, Falugi C, Fossa R, Genever P, Gallus L (2005). Interaction between organophosphate compounds and cholinergic functions during development.. Chem Biol Interact.

[r2] Ashwood P, Krakowiak P, Hertz-Picciotto I, Hansen R, Pessah I, Van de Water J. (2011). Elevated plasma cytokines in autism spectrum disorders provide evidence of immune dysfunction and are associated with impaired behavioral outcome.. Brain Behav Immun.

[r3] Ashwood P, Wills S, Van de Water J. (2006). The immune response in autism: a new frontier for autism research.. J Leukoc Biol.

[r4] Bader PL, Faizi M, Kim LH, Owen SF, Tadross MR, Alfa RW (2011). Mouse model of Timothy syndrome recapitulates triad of autistic traits.. Proc Natl Acad Sci USA.

[r5] Bailey A, Le Couteur A, Gottesman I, Bolton P, Simonoff E, Yuzda E (1995). Autism as a strongly genetic disorder: evidence from a British twin study.. Psychol Med.

[r6] Bauman ML, Kemper TL (2005). Neuroanatomic observations of the brain in autism: a review and future directions.. Int J Dev Neurosci.

[r7] Belloni V, Dessi-Fulgheri F, Zaccaroni M, Di Consiglio E, De Angelis G, Testai E (2011). Early exposure to low doses of atrazine affects behavior in juvenile and adult CD1 mice.. Toxicology.

[r8] Belmonte MK, Bourgeron T (2006). Fragile X syndrome and autism at the intersection of genetic and neural networks.. Nat Neurosci.

[r9] Biamonte F, Assenza G, Marino R, D’Amelio M, Panteri R, Caruso D (2009). Interactions between neuroactive steroids and reelin haploinsufficiency in Purkinje cell survival.. Neurobiol Dis.

[r10] Blatt GJ, Fitzgerald CM, Guptill JT, Booker AB, Kemper TL, Bauman ML (2001). Density and distribution of hippocampal neurotransmitter receptors in autism: an autoradiographic study.. J Autism Dev Disord.

[r11] Bolton PF, Carcani-Rathwell I, Hutton J, Goode S, Howlin P, Rutter M (2011). Epilepsy in autism: features and correlates.. The Br J Psychiatry.

[r12] Brannen KC, Devaud LL, Liu J, Lauder JM (1998). Prenatal exposure to neurotoxicants dieldrin or lindane alters *tert*-butylbicyclophosphorothionate binding to GABA_A_ receptors in fetal rat brainstem.. Dev Neurosci.

[r13] Breckenridge CB Holden L Sturgess N Weiner M Sheets L Sargent D 2009 Evidence for a separate mechanism of toxicity for the Type I and the Type II pyrethroid insecticides. Neurotoxicology 30 Suppl 1:S17 S31 19766671 10.1016/j.neuro.2009.09.002

[r14] Briz V, Molina-Molina JM, Sanchez-Redondo S, Fernandez MF, Grimalt JO, Olea N (2011). Differential estrogenic effects of the persistent organochlorine pesticides dieldrin, endosulfan, and lindane in primary neuronal cultures.. Toxicol Sci.

[r15] Brown DA (2010). Muscarinic acetylcholine receptors (mAChRs) in the nervous system: some functions and mechanisms.. J Mol Neurosci.

[r16] Burrow GN, Fisher DA, Larsen PR (1994). Maternal and fetal thyroid function.. N Engl J Med.

[r17] Buxbaum JD, Silverman JM, Smith CJ, Greenberg DA, Kilifarski M, Reichert J (2002). Association between a *GABRB3* polymorphism and autism.. Mol Psychiatry.

[r18] Campbell NA (1996).

[r19] Cao Z, Shafer TJ, Murray TF (2010). Mechanisms of pyrethroid insecticide-induced stimulation of calcium influx in neocortical neurons.. J Pharmacol Exp Ther.

[r20] Casida JE (2009). Pest toxicology: the primary mechanisms of pesticide action.. Chem Res Toxicol.

[r21] Centers for Disease Control and Prevention (2012). Prevalence of autism spectrum disorders—autism and developmental disabilities monitoring network, 14 sites, United States, 2008.. In: MMWR Surveill Summ.

[r22] Cheek AO, Kow K, Chen J, McLachlan JA (1999). Potential mechanisms of thyroid disruption in humans: interaction of organochlorine compounds with thyroid receptor, transthyretin, and thyroid-binding globulin.. Environ Health Perspect.

[r23] Colborn T. (2004). Neurodevelopment and endocrine disruption.. Environ Health Perspect.

[r24] Cook EH, Courchesne RY, Cox NJ, Lord C, Gonen D, Guter SJ (1998). Linkage-disequilibrium mapping of autistic disorder, with 15q11-13 markers.. Am J Hum Genet.

[r25] Corsini E, Liesivuori J, Vergieva T, Van Loveren H, Colosio C. (2008). Effects of pesticide exposure on the human immune system.. Hum Exp Toxicol.

[r26] Cossart R, Bernard C, Ben-Ari Y. (2005). Multiple facets of GABAergic neurons and synapses: multiple fates of GABA signalling in epilepsies.. Trends Neurosci.

[r27] Costa LG, Cole TB, Vitalone A, Furlong CE (2005). Measurement of paraoxonase (PON1) status as a potential biomarker of susceptibility to organophosphate toxicity.. Clin Chim Acta.

[r28] Crawley JN, Chen T, Puri A, Washburn R, Sullivan TL, Hill JM (2007). Social approach behaviors in oxytocin knockout mice: comparison of two independent lines tested in different laboratory environments.. Neuropeptides.

[r29] Curtis JT, Hood AN, Chen Y, Cobb GP, Wallace DR (2010). Chronic metals ingestion by prairie voles produces sex-specific deficits in social behavior: an animal model of autism.. Behav Brain Res.

[r30] D’Amelio M, Ricci I, Sacco R, Liu X, D’Agruma L, Muscarella LA (2005). Paraoxonase gene variants are associated with autism in North America, but not in Italy: possible regional specificity in gene–environment interactions.. Mol Psychiatry.

[r31] DeLorey TM, Sahbaie P, Hashemi E, Homanics GE, Clark JD (2008). Gabrb3 gene deficient mice exhibit impaired social and exploratory behaviors, deficits in non-selective attention and hypoplasia of cerebellar vermal lobules: a potential model of autism spectrum disorder.. Behav Brain Res.

[r32] Deth R, Muratore C, Benzecry J, Power-Charnitsky VA, Waly M (2008). How environmental and genetic factors combine to cause autism: A redox/methylation hypothesis.. Neurotoxicology.

[r33] Deutsch SI, Urbano MR, Neumann SA, Burket JA, Katz E (2010). Cholinergic abnormalities in autism: is there a rationale for selective nicotinic agonist interventions?. Clin Neuropharmacol.

[r34] Diamond B, Huerta PT, Tracey K, Volpe BT (2011). It takes guts to grow a brain: Increasing evidence of the important role of the intestinal microflora in neuro- and immune-modulatory functions during development and adulthood.. Bioessays.

[r35] Dolmetsch R. 2003 Excitation-transcription coupling: signaling by ion channels to the nucleus. Sci STKE 2003(166 pe4; doi: [Online 21 January 2003] 10.1126/stke.2003.166.pe4 12538881

[r36] Eddins D, Cerutti D, Williams P, Linney E, Levin ED (2010). Zebrafish provide a sensitive model of persisting neurobehavioral effects of developmental chlorpyrifos exposure: comparison with nicotine and pilocarpine effects and relationship to dopamine deficits.. Neurotoxicol Teratol.

[r37] Eskenazi B, Marks AR, Bradman A, Harley K, Barr DB, Johnson C (2007). Organophosphate pesticide exposure and neurodevelopment in young Mexican-American children.. Environ Health Perspect.

[r38] Ey E, Leblond CS, Bourgeron T (2011). Behavioral profiles of mouse models for autism spectrum disorders.. Autism Res.

[r39] Fatemi SH, Folsom TD, Reutiman TJ, Thuras PD (2009a). Expression of GABA_B_ receptors is altered in brains of subjects with autism.. Cerebellum.

[r40] Fatemi SH, Halt AR, Realmuto G, Earle J, Kist DA, Thuras P (2002). Purkinje cell size is reduced in cerebellum of patients with autism.. Cellular and molecular neurobiology.

[r41] Fatemi SH, Reutiman TJ, Folsom TD, Thuras PD (2009b). GABA_A_ receptor downregulation in brains of subjects with autism.. J Autism Dev Disord.

[r42] Franco R, Sanchez-Olea R, Reyes-Reyes EM, Panayiotidis MI (2009). Environmental toxicity, oxidative stress and apoptosis: menage a trois.. Mutat Res.

[r43] Gabbianelli R, Falcioni ML, Nasuti C, Cantalamessa F, Imada I, Inoue M (2009). Effect of permethrin insecticide on rat polymorphonuclear neutrophils.. Chem Biol Interact.

[r44] Galloway T, Handy R. (2003). Immunotoxicity of organophosphorous pesticides.. Ecotoxicology.

[r45] Geschwind DH (2011). Genetics of autism spectrum disorders.. Trends Cogn Sci.

[r46] Giulivi C, Zhang YF, Omanska-Klusek A, Ross-Inta C, Wong S, Hertz-Picciotto I (2010). Mitochondrial dysfunction in autism.. JAMA.

[r47] Goines P, Van de Water J. (2010). The immune system’s role in the biology of autism.. Curr Opin Neurol.

[r48] Goldner WS, Sandler DP, Yu F, Hoppin JA, Kamel F, Levan TD (2010). Pesticide use and thyroid disease among women in the Agricultural Health Study.. Am J Epidemiol.

[r49] Grandjean P, Landrigan PJ (2006). Developmental neurotoxicity of industrial chemicals.. Lancet.

[r50] Gupta RK, Schuh RA, Fiskum G, Flaws JA (2006). Methoxychlor causes mitochondrial dysfunction and oxidative damage in the mouse ovary.. Toxicol Appl Pharmacol.

[r51] Haas RH (2010). Autism and mitochondrial disease.. Dev Disabil Res Rev.

[r52] Halladay AK, Amaral D, Aschner M, Bolivar VJ, Bowman A, DiCicco-Bloom E (2009). Animal models of autism spectrum disorders: information for neurotoxicologists.. Neurotoxicology.

[r53] Hallmayer J, Cleveland S, Torres A, Phillips J, Cohen B, Torigoe T (2011). Genetic heritability and shared environmental factors among twin pairs with autism.. Arch Gen Psychiatry.

[r54] Hamilton SM, Spencer CM, Harrison WR, Yuva-Paylor LA, Graham DF, Daza RA (2011). Multiple autism-like behaviors in a novel transgenic mouse model.. Behav Brain Res.

[r55] Harrison PK, Falugi C, Angelini C, Whitaker MJ (2002). Muscarinic signalling affects intracellular calcium concentration during the first cell cycle of sea urchin embryos.. Cell Calcium.

[r56] Haviland JA, Butz DE, Porter WP (2009). Long-term sex selective hormonal and behavior alterations in mice exposed to low doses of chlorpyrifos *in utero*.. Reprod Toxicol.

[r57] Heijtz RD, Wang S, Anuar F, Qian Y, Bjorkholm B, Samuelsson A (2011). Normal gut microbiota modulates brain development and behavior.. Proc Natl Acad Sci USA.

[r58] Hertz-Picciotto I, Delwiche L. (2009). The rise in autism and the role of age at diagnosis.. Epidemiology.

[r59] Hertz-Picciotto I, Park HY, Dostal M, Kocan A, Trnovec T, Sram R (2008). Prenatal exposures to persistent and non-persistent organic compounds and effects on immune system development.. Basic Clin Pharmacol Toxicol.

[r60] Heuer L, Ashwood P, Schauer J, Goines P, Krakowiak P, Hertz-Picciotto I (2008). Reduced levels of immunoglobulin in children with autism correlates with behavioral symptoms.. Autism Res.

[r61] Heusinkveld HJ, Westerink RH (2012). Organochlorine insecticides lindane and dieldrin and their binary mixture disturb calcium homeostasis in dopaminergic PC12 cells.. Environ Sci Technol.

[r62] Kale M, Rathore N, John S, Bhatnagar D. (1999). Lipid peroxidative damage on pyrethroid exposure and alterations in antioxidant status in rat erythrocytes: a possible involvement of reactive oxygen species.. Toxicol Lett.

[r63] Karami-Mohajeri S, Abdollahi M. (2011). Toxic influence of organophosphate, carbamate, and organochlorine pesticides on cellular metabolism of lipids, proteins, and carbohydrates: a systematic review.. Hum Exp Toxicol.

[r64] Kaur P, Radotra B, Minz RW, Gill KD (2007). Impaired mitochondrial energy metabolism and neuronal apoptotic cell death after chronic dichlorvos (OP) exposure in rat brain.. Neurotoxicology.

[r65] Kenet T, Froemke RC, Schreiner CE, Pessah IN, Merzenich MM (2007). Perinatal exposure to a noncoplanar polychlorinated biphenyl alters tonotopy, receptive fields, and plasticity in rat primary auditory cortex.. Proc Natl Acad Sci USA.

[r66] Kim KH, Inan SY, Berman RF, Pessah IN (2009). Excitatory and inhibitory synaptic transmission is differentially influenced by two *ortho*-substituted polychlorinated biphenyls in the hippocampal slice preparation.. Toxicol Appl Pharmacol.

[r67] Kim KH, Pessah IN (2011). Perinatal exposure to environmental polychlorinated biphenyls sensitizes hippocampus to excitotoxicity *ex vivo*.. Neurotoxicology.

[r68] Lassiter TL, MacKillop EA, Ryde IT, Seidler FJ, Slotkin TA (2009). Is fipronil safer than chlorpyrifos? Comparative developmental neurotoxicity modeled in PC12 cells.. Brain Res Bull.

[r69] Laviola G, Adriani W, Gaudino C, Marino R, Keller F. (2006). Paradoxical effects of prenatal acetylcholinesterase blockade on neurobehavioral development and drug-induced stereotypies in reeler mutant mice.. Psychopharmacology.

[r70] Laviola G, Ognibene E, Romano E, Adriani W, Keller F. (2009). Gene–environment interaction during early development in the heterozygous *reeler* mouse: clues for modelling of major neurobehavioral syndromes.. Neurosci Biobehav Rev.

[r71] Le Couteur A, Bailey A, Goode S, Pickles A, Robertson S, Gottesman I (1996). A broader phenotype of autism: the clinical spectrum in twins.. J Child Psychol Psychiatry.

[r72] Lenz G, Avruch J. (2005). Glutamatergic regulation of the p70S6 kinase in primary mouse neurons.. J Biol Chem.

[r73] Levin ED, Addy N, Nakajima A, Christopher NC, Seidler FJ, Slotkin TA (2001). Persistent behavioral consequences of neonatal chlorpyrifos exposure in rats.. Dev Brain Res.

[r74] Levin ED, Chrysanthis E, Yacisin K, Linney E (2003). Chlorpyrifos exposure of developing zebrafish: effects on survival and long-term effects on response latency and spatial discrimination.. Neurotoxicol Teratol.

[r75] Levin ED, Timofeeva OA, Yang L, Petro A, Ryde IT, Wrench N (2009). Early postnatal parathion exposure in rats causes sex-selective cognitive impairment and neurotransmitter defects which emerge in aging.. Behav Brain Res.

[r76] Levin ED, Timofeeva OA, Yang L, Petro A, Ryde IT, Wrench N (2010). Early postnatal parathion exposure in rats causes sex-selective cognitive impairment and neurotransmitter defects which emerge in aging.. Behav Brain Res.

[r77] Li Q. (2007). New mechanism of organophosphorus pesticide-induced immunotoxicity.. J Nippon Med Sch.

[r78] Li X, Chauhan A, Sheikh AM, Patil S, Chauhan V, Li XM (2009). Elevated immune response in the brain of autistic patients.. J Neuroimmunol.

[r79] Liu J, Brannen KC, Grayson DR, Morrow AL, Devaud LL, Lauder JM (1998). Prenatal exposure to the pesticide dieldrin or the GABA_A_ receptor antagonist bicuculline differentially alters expression of GABA_A_ receptor subunit mRNAs in fetal rat brainstem.. Dev Neurosci.

[r80] Ma DQ, Whitehead PL, Menold MM, Martin ER, Ashley-Koch AE, Mei H (2005). Identification of significant association and gene–gene interaction of GABA receptor subunit genes in autism.. Am J Hum Genet.

[r81] Mackness B, Mackness MI, Arrol S, Turkie W, Durrington PN (1997). Effect of the molecular polymorphisms of human paraoxonase (PON1) on the rate of hydrolysis of paraoxon.. British journal of pharmacology.

[r82] Mariussen E, Fonnum F. (2006). Neurochemical targets and behavioral effects of organohalogen compounds: an update.. Crit Rev Toxicol.

[r83] McCauley JL, Olson LM, Delahanty R, Amin T, Nurmi EL, Organ EL (2004). A linkage disequilibrium map of the 1-Mb 15q12 GABA_A_ receptor subunit cluster and association to autism.. Am J Med Genet B Neuropsychiatr Genet.

[r84] Mense SM, Sengupta A, Lan C, Zhou M, Bentsman G, Volsky DJ (2006). The common insecticides cyfluthrin and chlorpyrifos alter the expression of a subset of genes with diverse functions in primary human astrocytes.. Toxicol Sci.

[r85] Ming X, Brimacombe M, Wagner GC (2007). Prevalence of motor impairment in autism spectrum disorders.. Brain Dev.

[r86] Moore PD, Yedjou CG, Tchounwou PB (2010). Malathion-induced oxidative stress, cytotoxicity, and genotoxicity in human liver carcinoma (HepG_2_) cells.. Environmental toxicology.

[r87] Morgan JT, Chana G, Pardo CA, Achim C, Semendeferi K, Buckwalter J (2010). Microglial activation and increased microglial density observed in the dorsolateral prefrontal cortex in autism.. Biol Psychiatry.

[r88] Morgan SL, Teyler TJ (1999). VDCCs and NMDARs underlie two forms of LTP in CA1 hippocampus in vivo.. J Neurophysiol.

[r89] Muhle R, Trentacoste SV, Rapin I (2004). The genetics of autism.. Pediatrics.

[r90] Murgia A, Zanardi I, Basso M, Deplano S, Falugi C, Prestipino G (2004). Electrophysyological and immunohistochemical studies of a organophospate pesticide on neuron K^+^ channels modulated by muscarinic receptor [Abstract].

[r91] Nakagami A, Koyama T, Kawasaki K, Negishi T, Ihara T, Kuroda Y (2011). Maternal plasma polychlorinated biphenyl levels in cynomolgus monkeys (*Macaca fascicularis*) affect infant social skills in mother–infant interaction.. Dev Psychobiol.

[r92] Neale BM Kou Y Liu L Ma’ayan A Samocha KE Sabo A 2012 Patterns and rates of exonic *de novo* mutations in autism spectrum disorders. Nature; doi:10.1038/nature11011 [Online 4 April 2012] PMC3613847 22495311

[r93] Onore C, Careaga M, Ashwood P. (2012). The role of immune dysfunction in the pathophysiology of autism.. Brain Behav Immun.

[r94] O’Roak BJ Vives L Girirajan S Karakoc E Krumm N Coe BP 2012 Sporadic autism exomes reveal a highly interconnected protein network of *de novo* mutations. Nature; doi:10.1038/nature10989 [Online 4 April 2012] PMC3350576 22495309

[r95] Park HY Hertz-Picciotto I Sovcikova E Kocan A Drobna B Trnovec T 2010 Neurodevelopmental toxicity of prenatal polychlorinated biphenyls (PCBs) by chemical structure and activity: a birth cohort study. Environ Health 9 51; doi: [Online 23 August 2010] 10.1186/1476-069X-9-51 20731829 PMC2939589

[r96] Park HY, Park JS, Sovcikova E, Kocan A, Linderholm L, Bergman A (2009). Exposure to hydroxylated polychlorinated biphenyls (OH-PCBs) in the prenatal period and subsequent neurodevelopment in eastern Slovakia.. Environ Health Perspect.

[r97] Pathak A, Sinha RA, Mohan V, Mitra K, Godbole MM (2011). Maternal thyroid hormone before the onset of fetal thyroid function regulates reelin and downstream signaling cascade affecting neocortical neuronal migration.. Cereb Cortex.

[r98] Patterson PH (2011). Brain–Immune Connections in Autism, Schizophrenia, and Depression. In: Infectious Behavior.

[r99] Peca J, Feliciano C, Ting JT, Wang W, Wells MF, Venkatraman TN (2011). Shank3 mutant mice display autistic-like behaviours and striatal dysfunction.. Nature.

[r100] Perry EK, Lee ML, Martin-Ruiz CM, Court JA, Volsen SG, Merrit J (2001). Cholinergic activity in autism: abnormalities in the cerebral cortex and basal forebrain.. Am J Psychiatry.

[r101] Persico AM, D’Agruma L, Maiorano N, Totaro A, Militerni R, Bravaccio C (2001). Reelin gene alleles and haplotypes as a factor predisposing to autistic disorder.. Mol Psychiatry.

[r102] Pessah IN, Cherednichenko G, Lein PJ (2010). Minding the calcium store: ryanodine receptor activation as a convergent mechanism of PCB toxicity.. Pharmacol Ther.

[r103] Pessah IN, Lein P (2008). Evidence for environmental susceptibility in autism. What we need to know about gene x environment interactions. In: Autism: Current Theories and Evidence, Part VI (Zimmerman AW, ed). Current Clinical Neurology.

[r104] Rathore M, Bhatnagar P, Mathur D, Saxena GN (2002). Burden of organochlorine pesticides in blood and its effect on thyroid hormones in women.. Sci Total Environ.

[r105] Roberts EM, English PB, Grether JK, Windham GC, Somberg L, Wolff C (2007). Maternal residence near agricultural pesticide applications and autism spectrum disorders among children in the California Central Valley.. Environ Health Perspect.

[r106] Rohlman DS, Anger WK, Lein PJ (2010). Correlating neurobehavioral performance with biomarkers of organophosphorous pesticide exposure.. Neurotoxicology.

[r107] Roman GC (2007). Autism: transient *in utero* hypothyroxinemia related to maternal flavonoid ingestion during pregnancy and to other environmental antithyroid agents.. J Neurol Sci.

[r108] Rooney AA, Matulka RA, Luebke RW (2003). Developmental atrazine exposure suppresses immune function in male, but not female Sprague-Dawley rats.. Toxicol Sci.

[r109] Rosenberg RE, Law JK, Yenokyan G, McGready J, Kaufmann WE, Law PA (2009). Characteristics and concordance of autism spectrum disorders among 277 twin pairs.. Arch Pediatr Adolesc Med.

[r110] Rossignol DA, Frye RE (2012). Mitochondrial dysfunction in autism spectrum disorders: a systematic review and meta-analysis.. Mol Psychiatry.

[r111] Rubenstein JL, Merzenich MM (2003). Model of autism: increased ratio of excitation/inhibition in key neural systems.. Genes Brain Behav.

[r112] Samaco RC, Hogart A, LaSalle JM (2005). Epigenetic overlap in autism-spectrum neurodevelopmental disorders: MECP2 deficiency causes reduced expression of UBE3A and GABRB3.. Hum Mol Genet.

[r113] Sanders SJ Murtha MT Gupta AR Murdoch JD Raubeson MJ Willsey AJ 2012 *De novo* mutations revealed by whole-exome sequencing are strongly associated with autism. Nature; doi:10.1038/nature10945 [Online 4 April 2012] PMC3667984 22495306

[r114] Scharf ME (2003). Neurological effects of insecticides.

[r115] Schuh RA, Kristian T, Gupta RK, Flaws JA, Fiskum G (2005). Methoxychlor inhibits brain mitochondrial respiration and increases hydrogen peroxide production and CREB phosphorylation.. Toxicol Sci.

[r116] Schuh RA, Richardson JR, Gupta RK, Flaws JA, Fiskum G (2009). Effects of the organochlorine pesticide methoxychlor on dopamine metabolites and transporters in the mouse brain.. Neurotoxicology.

[r117] Shi X, Gu A, Ji G, Li Y, Di J, Jin J (2011). Developmental toxicity of cypermethrin in embryo–larval stages of zebrafish.. Chemosphere.

[r118] Sigala S, Zoli M, Palazzolo F, Faccoli S, Zanardi A, Mercuri NB (2007). Selective disarrangement of the rostral telencephalic cholinergic system in heterozygous reeler mice.. Neuroscience.

[r119] Silverman JL, Yang M, Lord C, Crawley JN (2010). Behavioural phenotyping assays for mouse models of autism.. Nature Rev Neurosci.

[r120] Sinagra M, Gonzalez Campo C, Verrier D, Moustie O, Manzoni OJ, Chavis P (2008). Glutamatergic cerebellar granule neurons synthesize and secrete reelin in vitro.. Neuron Glia Biol.

[r121] Slotkin TA, Bodwell BE, Levin ED, Seidler FJ (2008). Neonatal exposure to low doses of diazinon: long-term effects on neural cell development and acetylcholine systems.. Environ Health Perspect.

[r122] Soderlund DM (2012). Molecular mechanisms of pyrethroid insecticide neurotoxicity: recent advances.. Arch Toxicol.

[r123] Splawski I, Timothy KW, Sharpe LM, Decher N, Kumar P, Bloise R (2004). Ca_V_1.2 calcium channel dysfunction causes a multisystem disorder including arrhythmia and autism.. Cell.

[r124] Stafstrom CE, Hagerman PJ, Pessah IN (2010). Epilepsy in autism spectrum disorders.. Epilepsia.

[r125] Stankiewicz P, Lupski JR (2010). Structural variation in the human genome and its role in disease.. Annu Rev Med.

[r126] Steffenburg S, Gillberg C, Hellgren L, Andersson L, Gillberg IC, Jakobsson G (1989). A twin study of autism in Denmark, Finland, Iceland, Norway and Sweden.. J Child Psychol Psychiatry.

[r127] Tabuchi K, Blundell J, Etherton MR, Hammer RE, Liu X, Powell CM (2007). A neuroligin-3 mutation implicated in autism increases inhibitory synaptic transmission in mice.. Science.

[r128] Tuchman R, Cuccaro M. (2011). Epilepsy and autism: neurodevelopmental perspective.. Curr Neurol Neurosci Rep.

[r129] U.S. Department of Agriculture (2006). Pest Management Practices. In: Agricultural Resources and Environmental Indicators (Wiebe K, Gollehon N, eds). 2006 ed. EIB-16.. http://www.ers.usda.gov/publications/arei/eib16/Chapter4/4.3/.

[r130] U.S. Environmental Protection Agency (2011). Basic Information about Polychlorinated Biphenyls (PCBs) in Drinking Water.. http://water.epa.gov/drink/contaminants/basicinformation/polychlorinated-biphenyls.cfm.

[r131] Vargas DL, Nascimbene C, Krishnan C, Zimmerman AW, Pardo CA (2005). Neuroglial activation and neuroinflammation in the brain of patients with autism.. Ann Neurol.

[r132] Varju P, Katarova Z, Madarasz E, Szabo G. (2001). GABA signalling during development: new data and old questions.. Cell Tissue Res.

[r133] Watanabe M, Maemura K, Kanbara K, Tamayama T, Hayasaki H. (2002). GABA and GABA receptors in the central nervous system and other organs.. Int Rev Cytol.

[r134] Wegiel J, Kuchna I, Nowicki K, Imaki H, Marchi E, Ma SY (2010). The neuropathology of autism: defects of neurogenesis and neuronal migration, and dysplastic changes.. Acta Neuropathologica (Berlin).

[r135] West AE, Griffith EC, Greenberg ME (2002). Regulation of transcription factors by neuronal activity.. Nature Rev Neurosci.

[r136] Williams MK, Rundle A, Holmes D, Reyes M, Hoepner LA, Barr DB (2008). Changes in pest infestation levels, self-reported pesticide use, and permethrin exposure during pregnancy after the 2000–2001 U.S. Environmental Protection Agency restriction of organophosphates.. Environ Health Perspect.

[r137] Winneke G. (2011). Developmental aspects of environmental neurotoxicology: lessons from lead and polychlorinated biphenyls.. J Neurol Sci.

[r138] Woodruff TJ, Zota AR, Schwartz JM (2011). Environmental chemicals in pregnant women in the United States: NHANES 2003–2004.. Environ Health Perspect.

[r139] Yan Y, Yang Y, You J, Yang G, Xu Y, Huang N (2011). Permethrin modulates cholinergic mini-synaptic currents by partially blocking the calcium channel.. Toxicol Lett.

[r140] Yang D, Howard A, Bruun D, Ajua-Alemanj M, Pickart C, Lein PJ (2008). Chlorpyrifos and chlorpyrifos-oxon inhibit axonal growth by interfering with the morphogenic activity of acetylcholinesterase.. Toxicol Appl Pharmacol.

[r141] Yang D, Lauridsen H, Buels K, Chi LH, La Du J, Bruun DA (2011). Chlorpyrifos-oxon disrupts zebrafish axonal growth and motor behavior.. Toxicol Sci.

[r142] Zhang X, Daugherty SL, de Groat WC (2010). Activation of CaMKII and ERK1/2 contributes to the time-dependent potentiation of Ca2^+^ response elicited by repeated application of capsaicin in rat DRG neurons.. Am J Physiol Regul Integr Comp Physiol.

[r143] Zimmermann MB (2007). The adverse effects of mild-to-moderate iodine deficiency during pregnancy and childhood: a review.. Thyroid.

